# Vascularized Composite Allografts: Procurement, Allocation, and Implementation

**DOI:** 10.1007/s40472-014-0025-6

**Published:** 2014-07-03

**Authors:** Axel Rahmel

**Affiliations:** Deutsche Stiftung Organtransplantation, Deutschherrnufer 52, Frankfurt am Main, 60594 Frankfurt, Germany

**Keywords:** Transplantation, Vascularized composite allografts, Hand, Face, Procurement, Allocation, Ethics, Policy

## Abstract

Vascularized composite allotransplantation is a continuously evolving area of modern transplant medicine. Recently, vascularized composite allografts (VCAs) have been formally classified as ‘organs’. In this review, key aspects of VCA procurement are discussed, with a special focus on interaction with the procurement of classical solid organs. In addition, options for a matching and allocation system that ensures VCA donor organs are allocated to the best-suited recipients are looked at. Finally, the different steps needed to promote VCA transplantation in society in general and in the medical community in particular are highlighted.

## Introduction – Vascular Composite Allografts (VCAs) as Organs

Vascularized composite allotransplantation refers to the transfer of a vascularized human body part containing multiple tissue types (skin, muscle, bone, nerves, and blood vessels) as an anatomical and/or structural unit from a human donor to a human recipient. The first reported unilateral hand transplantation was performed in Ecuador in 1963, but the transplant had to be removed within two weeks due to acute rejection [1, 2]. The first successful hand transplantation after the introduction of cyclosporine followed 35 years later in 1998 in France [3, 4] and started a new era of vascularized composite allotransplantation. In 2005, the first face transplantation was performed in Lyon [5]; since then the number of face transplants has increased [6]. This special category of transplants is localized at the border between tissue and organ transplantation. The term composite tissue allotransplantation (CTA) used in the past reflects that it was often considered as a special type of tissue transplantation [7]. Several reports looking in-depth at limb and face transplantation made it clear that vascularized composite allotransplantation is in central aspects more similar to organ than to tissue transplantation [7–12]. After careful evaluation involving the transplant community and the general public, the United States Department of Health and Human Services recently published its decision to recognize vascularized composite allografts (VCAs) as organs and defined VCAs based on nine criteria (Table 1) [13]. A non-exclusive list of body parts that meet the definition of VCAs implemented in the US rule include face, limbs (e.g., arm, hand), larynx, and abdominal wall [14].

**Table 1:** Defining criteria for a vascularized composite allograft (VCA)

VCA means a body part:
1. That is vascularized and requires blood flow by surgical connection of blood vessels to function after transplantation
2. Containing multiple tissue types
3. Recovered from a human donor as an anatomical/structural unit
4. Transplanted into a human recipient as an anatomical/structural unit
5. Minimally manipulated (i.e., processing that does not alter the original relevant characteristics of the organ relating to the organ’s utility for reconstruction, repair, or replacement)
6. For homologous use (the replacement or supplementation of a recipient’s organ with an organ that performs the same basic function or functions in the recipient as in the donor)
7. Not combined with another article such as a device
8. Susceptible to ischemia and, therefore, only stored temporarily and not cryopreserved
9. Susceptible to allograft rejection, generally requiring immunosuppression that may increase infectious disease risk to the recipient^a^

Adapted from http://federal.eregulations.us/fr/notice/7/3/2013/2013-15731 [13].

^a^ In exceptional cases (identical twins or sharing of highly concordant histocompatibility matching markers), the recipient might not require any immunosuppression.

A similar development regarding the categorization of VCAs took place in Europe. In *Directive 2004/23/EC of the European Parliament and of the Council on setting standards of quality and safety for the donation, procurement, testing, processing, preservation, storage and distribution of human tissues and cells* (European Union [EU] tissue and cell directive) [15], organs were defined as “a differentiated and vital part of the human body, formed by different tissues, that maintains its structure, vascularization and capacity to develop physiological functions with an important level of autonomy”. As VCAs are in general not a ‘vital’ part of the body, it was initially concluded by the National Competent Authorities (CA) of the EU Member States in charge of tissues and cell transplantation that VCAs could not be classified as organs [11, 16]. In 2010, the European Parliament and the Council released *Directive 2010/45/EU on standards of quality and safety of human organs intended for transplantation* (EU organ directive) [17]. In this directive, the definition of an organ has been slightly but importantly modified among other things by removing the word ‘vital’ from the definition. This change took place primarily in order to include kidneys and pancreata in the definition of an ‘organ’. Taking into account this new definition and recent developments in the transplantation of VCAs, the National CA and the CA in charge of tissues and cell transplantation independently discussed whether VCAs fall under the ‘tissue and cell’ or under the ‘organ’ directive. The common understanding of both CA groups is that VCAs fall under the organ directive [18]. The impact of the new categorization of VCAs and its relation to the ‘classical’ solid organ transplantation will be the focus of this brief review.

## Procurement

### Consent

A major prerequisite for the procurement of a VCA is the consent by the donor or next of kin. There is general understanding that although VCAs will now be classified as organs, currently existing consent to ‘organ donation’ as documented in donor registries or on donor cards does in general not cover consent to VCA donation. As such, the importance of a transparent and explicit consent for VCA donation is highlighted in the document from the US Department of Health and Human Services classifying VCAs as organs. This will include an adaptation of donor forms and the approach to the family, so that it is clear whether the consent covers the procurement and transplantation of VCA and whether certain body parts are excluded from this consent. In the reports on VCA transplantation from European countries, it was made clear that explicit consent was required from the family for VCA donation, even in countries with presumed consent legislation. In fact, the consent process, especially for facial allograft donation, has shown to be a major challenge [19•]. There has repeatedly been concern that asking for consent for VCA donation might negatively influence the willingness to donate classical solid organs [20, 21]. This aspect has to be taken into account in future public campaigns on organ donation and especially when approaching the family of a potential organ donor. Asking for VCA must not hamper the consent and donation process for potentially life-saving solid organs; therefore, it is generally recommended that a request for VCA transplantation shall only be made after approval for organ donation has been granted, and only in carefully selected cases.

Intercultural variation regarding the acceptance of facial transplantation has been reported. In addition, the willingness to accept a face transplant was higher (68 %) than the willingness to donate one’s face after death (41 %) in one investigation [22••]. Religious beliefs also play a role and must be taken into consideration [23], both when planning public campaigns that include VCA transplantation and when approaching the family of a possible donor.

### Identification of a Tissue Donor, Donor Characterization, and Donor Management

Vascularized composite allotransplantation is typically not a life-saving treatment; therefore, careful donor selection is of upmost importance to prevent the transmission of any unwanted disorders. A detailed work-up of the donor is essential [24–26]. As brain death, especially in younger donors, often occurs as a consequence of trauma, damage to the VCA to be procured, including fracture of the bones, has to be ruled out [27]. The donor has to be well characterized with radiomorphometric studies and detailed determination of the vessel status [27, 28•]. In this context, it is recommended to remove arterial lines and to perform an angiogram to see whether vessel patency and perfusion is impaired in case of doubt. [29].

### Surgical Procedure

The surgical details of the procurement procedure vary from one type of VCA to another and are beyond the scope of this review. Extensive experimental data have been gathered for the different types of VCA procurement and transplantation and several reports summarizing current clinical practice have been published [14, 19•, 28•, 30, 31, 32••, 33–39, 40••, 41]. This review will focus on upper-limb and face procurement and only on those elements important for the interaction with the procurement of other classical solid organs.

Gordon developed a classification system for VCA procurement and transplantation that is based on the relative complexity of the procedure. It shows that upper extremity and face transplantation are characterized by a high degree of complexity, substantially more complex than, for example, abdominal wall transplantation and only outmatched by the complexity of combined VCA transplantation [42••, 43].

An important aspect for face replantation and transplantation is the knowledge of facial angiosomes first described by Houseman et al. in 2000 [44]. This has direct impact on the technique of procuring a face [32••, 33]. Based on the underlying defect in the recipient, facial allograft recovery will vary substantially from case to case [19•]. If no bone defects exist, only skin and soft tissue of the face need to be procured, typically by dissecting in the subgaleal, sub-superficial muscular aponeurotic system (sub-MSAS) [32••, 34]. In case of accompanying bone defects, soft tissue and bony structures must be procured as an osteocutaneous composite flap in a subperiosteal plane [32••]. The fine-tuning of the VCA with removal of excess tissues, tendons and bones, nerves and vessels to precisely match donor and recipient is typically done after transport of the VCA, on the side table at the recipient center.

Although rejection in a sentinel skin graft is not necessarily representative of rejection in the main VCA, the use of a sentinel skin allograft to allow skin biopsies without affecting the VCA has been used [45].

Minimizing immunosuppression to reduce side effects related to this therapy (tumor, infection, kidney failure) is an important goal and it is a focus of the field to understand the VCA specificities and potential treatment options [46].

### Coordination of Procurement With Other Organs

Similar to the fact that the question of consent for VCA procurement must not negatively influence solid organ procurement, there is general agreement that procurement of VCA must not hamper the retrieval of other potentially life-saving organs. Well structured time planning is essential. Hand and forearm should be harvested prior to solid organs if possible to prevent impaired hand perfusion and prolongation of ischemia time [27, 47]. This approach is of limited influence on solid organ procurement as the surgical procedure in general takes less than 30 minutes [28•] and is typically done under tourniquet, which stays on the donor arm until the end of the procurement. If the other arm is also being removed, the procedure gets more complex and good coordination with the thoracic organ team(s) is mandatory [48].

For face transplantation, multiple vessels, nerves, muscles, and osseous structures have to be identified and dissected carefully, so the procurement is much more complex and typically takes about 7–12 hours. In some cases, face procurement has taken as long as 22 hours [49••, 50]. That length of time has been considered too long for life-saving organs to wait by some teams. A group from Spain reported that in a hemodynamically unstable donor, all life-saving organs were procured first. In a second step, the face was procured in a bloodless-cold field to avoid the risk of losing non-VCA organs and reduced warm ischemia time [50].

If the face is procured first, coordination with the solid organ teams is of upmost importance, due to the fact that the donor might become unstable during the long preparatory phase. In case a ‘face first’ approach is chosen, the following three strategies regarding access to the thoracic and/or abdominal structures can be distinguished depending on donor stability, risks associated with the face procurement, and local preferences [29, 49••, 50].

1. If the donor is unstable or the VCA procurement is a high-risk procedure, the thoraco-abdominal incision could take place at the beginning of the procurement procedure or be triggered by signs of hemodynamic deterioration of the donor.
2. In any phase of face harvesting with an elevated risk of blood loss it could be an option to have the solid organ teams remaining on stand-by in-house, ideally already scrubbed. Such an approach might pose quite a burden on the explant teams for the solid organs, especially if non-local teams are involved.
3. Often the arrival of the solid organ teams in the operating room (OR) and solid organ retrieval are postponed until shortly prior to the planned VCA explantation.

Independent of the selected individual strategy, the aim has to be to prevent solid organ loss.

Prior to the procurement of the face, typically a tracheostomy is performed because orotracheal intubation might hamper the surgical procedure [40••]. This has led to concern with some lung retrieval teams [50], although so far no negative impact of tracheostomizing the donor on lung procurement has been reported. In any case, this question has to be discussed and agreed upon among the involved teams, underlining the need for close cooperation in case of VCA procurement.

### Donor Reconstruction

The VCA recovery team is responsible for donor reconstruction, which can pose a special challenge in face transplantation. Creating an adequate aesthetic prosthesis that can be attached to the donor after procurement can be quite demanding, especially in case of face procurement [27]. Several techniques to create such masks to reconstruct the face after procurement have been described [50]. This approach preserves donor’s dignity, an aspect important not only for the donor family but also for all others involved in the donation process and thereby for the acceptance of face transplantation in society in general [19•, 21].

## Allocation

The organ allocation process has to be transparent, objective and reliable. Each step in the allocation process should be well documented to allow full accountability towards the transplant community and the general public. The underlying allocation rules shall be based on sound medical judgment and are typically specific for each organ type. They shall seek to achieve the best use of the organs, avoiding wasting of organs and futile transplantation. Allocation of organs typically takes place in two steps:

Step 1 Selection: identifying those patients that are at all suitable for a specific organ among all patients on the waiting list.
Step 2 Ranking: determining the allocation sequence among all suitable recipients.

### Selection Criteria for the Identification of Suitable Recipients

There are a few general selection criteria: the blood group of donor and recipient have to be compatible. While it is desirable to have a good immunological match between donor and recipient, HLA mismatches between donor and recipient are not a contraindication to transplantation [27, 51]. This is especially true as long as the number of donors and recipients is limited. Strict requirements regarding HLA matching between donor and recipient might result in a loss of donor organs. While a high number of HLA mismatches between donor and recipient is not a contraindication to transplantation, it could be included in the risk/benefit estimate when deciding about accepting an organ offer for an individual recipient [12, 29, 52, 53]. Patients should be screened for the presence of HLA antibodies at time of listing and after events that could result in immunization. In the event of an organ offer with known donor-specific antibodies, a transplantation is not recommended. A pre-operative lymphocytotoxic crossmatch is considered mandatory and should be negative [19•, 41, 54••].

Next to these general considerations, other prerequisites for the selection of suitable recipients have been proposed. Age difference of donor and recipient should be limited. Results of a computer simulation suggested that it is acceptable when the donor is between 2 decades younger and 1 decade older than the recipient [55]. In France, the age difference between donor and recipient should be limited to 10 years. Another approach could be that for each recipient the acceptable age range is defined individually, taken recipient wishes into account, and respected in the allocation process [56]. There has been some debate whether gender difference between donor and recipient poses a problem for VCA transplantation. At least in the case of hand transplantation, gender mismatch has been considered acceptable [29, 32••].

Instead of using surrogate parameters like age and gender for matching, specific phenotypic matching based on size, especially concerning bones, color and texture of the skin, and soft tissue features has been recommended by several authors [25, 32••, 57]. Organ-specific parameters have to be defined to allow adequate anthropometric measurements and the corresponding matching. For example, only limited differences between donor and recipient regarding key parameters like craniofacial size can be tolerated in face transplantation. Differences in the range of 9–14 % yielded already unacceptable results in an experimental setting [32••]. Carefully planned studies with large sample sizes together with a detailed collection of the donor and recipient data are needed to identify the most relevant anthropometric measures and their specific compatibility ranges [32••, 35, 44].

Next to skin color and texture, soft tissue features like the cartilaginous nose, the lips, and the eyebrows are also important in order to let the recipient’s face or limbs look similar to the previous native situation after transplantation. When determining acceptable differences between donor and recipient it probably has to be taken into account that conventional dermatologic techniques and/or conventional makeup could be used to mitigate modest differences between donor and recipient [19•, 28•]. Of course, acceptance criteria may also vary from recipient to recipient based on individual preferences and the urgency of transplantation. There might be patients that would only accept a VCA if it closely matches their own skin color and texture, other patients might be less selective in this regard. Therefore, individual patient-specific profiles regarding these phenotypic donor characteristics might be used in allocation in the future. This is of special relevance if the exchange of VCA across different organ procurement organizations (OPOs) is considered. Of course, patients have to be made aware that the more specific and restrictive the profile, the lower the probability of finding a suitable recipient quickly.

The respective donor characterization and reporting to the allocation organization have to be based on these criteria and can then be used in matching. This might include the use of standardized color cards and standardized rules and techniques to determine donor size [19•, 29, 58••].

### Determination of the Allocation Sequence

#### Possible Allocation Factors

Once suitable recipients have been identified, it is necessary to determine the allocation sequence. Different criteria might be used for this.

Outcome of transplantation could be one important criterion. HLA matching between donor and recipient could be taken into account in allocation, because better matching might positively influence long-term results of transplantation due to less acute and chronic rejection. In kidney transplantation it has been shown that with better matching there is a reduced need for immunosuppression, thus mitigating the related short- and long-term complications (renal insufficiency, infections, tumor disease, etc.) [59]. Therefore, this approach has also been suggested for VCA transplantation [24, 52, 60–62]. Considering the fact that a clear cut-off point regarding the maximal number of acceptable HLA mismatches between donor and recipient has never been shown in solid organ transplantation, and that both the donor and recipient pool for VCA transplantation are currently very small, adequate HLA matching between donor and recipient will not be achievable or necessary. But, with otherwise similarly suited recipients, better HLA matching could help with the prioritization of recipients.

Recipients with a high level of donor-specific antibodies should be avoided to reduce the risk of graft loss both in the early phase after transplantation and later due to chronic rejection with graft vasculopathy [29].

Next to patient and graft survival, the expected functional and cosmetic transplant results which are influenced by the quality of phenotypic matching between donor and recipient could play a role in allocation. Based on the phenotypic characteristics of donor and recipients, the best match in size, (skin) pigmentation, and texture could be identified and given priority [27, 28•, 32••].

Urgent patients, for example patients with arteriovenous malformation with severe bleeding waiting for a face transplant or patients with severe psychological distress, could be given priority.

For highly immunized patients or patients waiting for a combined transplant, it is often extremely difficult to find a suitable donor and expected waiting times are very long. As a compensation for this disadvantage these patients could be given priority in allocation if a suitable donor for them is available.

Taking the aspect of general fairness into consideration, waiting time could be used for prioritization, especially as VCA transplantation is not a life-saving procedure.

Currently, VCAs are typically allocated locally or regionally (first). One argument in favor of this approach is the expected resulting shorter ischemic time. Some experimental studies showed that extended ischemic time increases tissue damage and has a negative impact on long-term structural and functional outcome, in others an effect of ischemic time on VCA function was not shown [51]. As such, ischemic time can be seen as a surrogate marker for the success of transplantation. Although a clear upper limit for the acceptable ischemic time for VCA can’t be defined currently, it seems reasonable to keep ischemic time as short as possible. The ischemic time is mainly dependent on the transport time and the surgical time needed until start of reperfusion. As transport time can be influenced more easily then the time needed for the complex surgery, it has been suggested to keep the transport time shorter than 4 hours [19•, 29, 51, 63]. Next to the expected shorter ischemic time there are other arguments in favor of local or regional allocation of VCA. In most countries, the abdominal organs are typically procured by a local/regional team. Therefore, it would be possible to prepare and train the complex coordination between the different explant teams as described above well in advance. In general, the logistical challenges would be less because communication lines and travel distances between donor hospital and transplant center are short. For example, the timely preparation of the necessary prostheses for the donor would be more feasible with local allocation. In addition, local or regional allocation would allow a relationship to develop between the VCA transplant center and possible donor hospitals. It has been shown that this approach has a positive impact on reporting possible VCA donors and on the perception of the VCA procurement procedure by the personnel in the donor hospital.

#### Options for Interaction of the Different Allocation Factors to Create a Match List

**Fig. 1:**
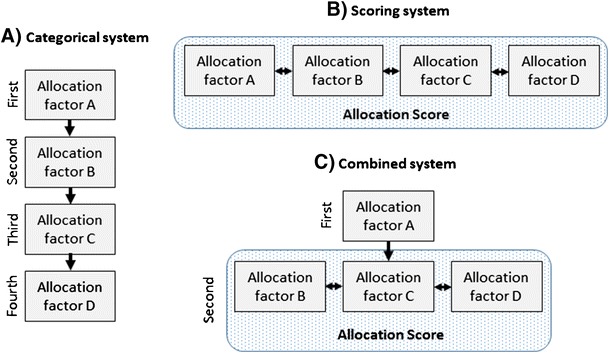
Schematic representation of different allocation models

Regarding the interaction of the different allocation factors for determining the allocation sequence, different strategies can be distinguished (Fig. 1):

A) Categorical or tier-based system
  The different allocation criteria are used in a sequential manner starting with the most important one. All recipients are subdivided into two or more categories based on the first criterion and each of the resulting categories determines a different priority level. If there is only one recipient in the top priority tier, he/she will get the organ offer. If there is more than one recipient in that category, the next allocation criterion will be used to further subdivide the remaining patients. For example, as a first step, recipients could be subdivided into urgent and elective patients. If there is more than one urgent patient, local patients could get priority in this group. If there is more than one urgent local patient, HLA matching or phenotypic matching could further subdivide groups. As a final tie-breaker, waiting time could be used. While this system is simple to understand and to implement, it has the major disadvantage that there is a strict hierarchy of the criteria from the most to the least important one. This problem is avoided in the score-based system.
B) Score-based system
  Here all identified allocation criteria are used at the same time to determine the allocation sequence. For each criterion, an individual point value is assigned. The total score is calculated based on a mathematical algorithm using all individual values of the different criteria. A very simple score system could be the sum of the values for the different allocation criteria (some points for each element of the phenotypic matching, for HLA matching, waiting time, distance, or expected ischemic time, etc.). However, more complex scores could also be used to reflect the interaction of the different allocation factors (a typical example of such a scoring system is the MELD [model of end-stage liver disease] score used in liver allocation or the Lung Allocation Score [LAS] used in lung allocation in some countries).
C) Combined system
  Often a combination of both systems is used. Patients are subdivided into two or more main tiers and within each tier the allocation sequence is based on a score calculated using other criteria. For example, patients could be subdivided into high-urgent patients and patients with normal urgency. In that case, high-urgent patients would always have priority over patients with standard urgency. Within each tier, the allocation sequence would be based on a score using the other allocation factors.

### Practical Consequences for VCA Allocation

VCA transplantation is currently very much based on ad hoc arrangements, typically within an OPO or a region thereof. At present, VCA transplantation is more about looking for a donor for an individual patient than about assigning a VCA from a donor to the most suitable recipient. This approach is quite similar to the situation in the early days of solid organ transplantation. Logistical aspects are – especially in this early phase of setting up VCA transplantation – a strong argument in favor of this local allocation system because donor hospitals have to be involved and the coordination with other teams has to be agreed upon [25]. On the other hand, this system is not very effective. Sharing of VCAs over longer distances would have the advantage of a larger donor and recipient pool and therefore would allow better matching, yielding better transplant results [64•]. A prerequisite for sharing is an integrated database containing the specific immunologic and anatomical criteria needed for selection of recipients based on the donor characteristics. There are some early cases of successful longer distance exchange of VCAs [54••] in the US showing the feasibility of this approach. If the number of recipients on the waiting list for a VCA increases, implementation of transparent allocation rules will anyway be necessary. A combination of categorical and scoring systems might the best solution to start with. For example, in France, sensitized patients, urgent cases (arteriovenous malformation with severe bleeding for face transplantation), or combined VCA (face + arm) transplantations are given priority [29]. Within the groups of patients with high and standard priority, the allocation sequence can then be determined with a scoring system. Most probably the role of local/regional allocation will be controversial in the beginning. The more VCA transplantation becomes daily practice, the less relevant the question of local or regional allocation will become.

## Prerequisites for the Implementation

VCA transplantation is currently a low volume area with all the related challenges of training donor hospitals, procurement teams, and transplant centers. Together with the aforementioned aspects of procurement and allocation, several prerequisites for stimulating and spreading VCA transplantation in general and setting up individual transplant programs can be derived.

There should be a policy for informing the general public about the need and the benefits of VCA transplantation. At present, the possible demand for VCA transplantation is unclear. Approximately 100,000 upper-extremity amputees are living in the US at the moment [65], but of course the selection of recipients has to be based on a careful risk–benefit analysis, taking alternative therapies including modern prostheses into account. Currently, only a minority of these patients are candidates for VCA transplantation [20, 27, 66, 67]. A similar strict selection process will be necessary for all other types of VCA transplantation. For these candidates, VCA transplantation could have a major positive impact on their daily life, allowing social reintegration [43, 60, 68]. Public campaigns regarding VCA donation should be carried out in a balanced way to educate about consent for VCA without lowering the total consent rates for organ donation, as the impact of establishing a VCA transplant program on organ donation is currently unknown [20, 25].

Educational meetings with donor hospitals and OPOs are necessary to allow full understanding of the complex donor procedure – VCA transplantations must not come as a surprise to the donor hospital. OPOs have to be especially trained and informed regarding how to approach the family and ask for informed consent. A VCA-tailored algorithm should be used when approaching all potential donor families for VCA, especially face transplantation [58••]. Vital organs (such as heart, lung, liver, kidney, and pancreas) are always discussed first, so that the request for VCA donation does not interfere with requesting life-saving organs [42••]. It has been reported that showing the need for VCA transplantation by presentation of eligible candidates pursuing VCA transplantation [42••], and how the donated VCA would help with reintegration into society [25], is a strong motivation for employees in the donor hospitals.

Organ allocation organizations have to discuss and develop matching criteria and allocation rules [69••]. Based on these, the IT infrastructure has to be adapted to allow central registration of patients and donors with all relevant matching criteria. Some of them (morphometric information, color etc.) will be completely new and different from current practice so that procedures for standardized reporting have to be established in order to allow reasonable matching.

Setting up a VCA transplant program is a complex and challenging process for a transplant center. Several prerequisites have to be met to create a successful program. Requirements for the development of a hand transplant program have been described [27, 64•, 70], the challenges for a face transplant program might even be more pronounced [19•, 25, 42••, 69••, 71–74]. The composition of and close cooperation within the transplant team are key for the success of a new program. A microsurgical-trained plastic surgeon, a transplant expert, a transplant immunologist, an infectious disease expert, a social worker, an ethicist, and a transplant psychiatrist/psychologist are at the core of the team. ICU staff, anesthesiology, OR manager, physical therapy, and rehabilitation have to be closely involved, too. It has been suggested to establish the role of a (face) transplant coordinator involved in all steps from assessing potential candidates, providing the patients with the necessary information, and coordinating the procurement/transplantation process, to supporting follow-up care. The surgical procedure itself should be prepared carefully including a series of mock, fresh-cadaver training transplants.

Clear rules for the VCA procurement team including the interaction and cooperation with the other procurement teams have to be developed. It is highly recommended to establish common guidelines per type of VCA for all procurement teams. This would make procurement outside the local OPO more easy and feasible. In an interim period, it might be an option that a procurement team visiting a donor hospital outside their own OPO is joined in a type of rendezvous system by a VCA procurement expert from the local (VCA) transplant team for organizational support and to guarantee smooth cooperation with the donor center. This might even include support in donor reconstruction.

As long as there are only a few VCA transplant centers, recipients might undergo transplantation in a center quite distant from home. Therefore, cooperation with special training of external doctors to allow local long-term follow-up has to be setup [75].

A new VCA transplant program must only start with its first transplants after formal approval by the responsible authorities [10, 21]. This implies that transparent guidelines and criteria have to be developed, and based upon these it will be judged whether a center is ready for VCA transplantation. Continuous monitoring of the VCA transplant activities and related complications and long-term outcomes is essential for continuous improvement of VCA transplantation. The current (voluntary) reporting to the International Registry of Hand and Composite Tissue Transplantation is not sufficient. A significant number of VCA transplant recipients are never reported to the registry or follow-up is missing. In an emerging field that has been experimental until recently, this is certainly not desirable and mandatory reporting of all transplants including follow-up should be aimed at [76].

## Conclusions

VCA transplantation is a still small but promising section of organ transplantation. The success and possible further growth of this new field will depend on several factors: a coordinated team approach involving multiple disciplines in the VCA transplant center, and excellent cooperation with the respective organ procurement organization as well as transparent interaction with the other solid organ procurement teams. This medical approach has to be accompanied by thorough, well planned public information on all aspects of VCA transplantation, because willingness to donate VCA is the most important but also the most critical prerequisite for expanding the donor pool. The more common VCA transplantation becomes, the more allocation will move away from the current primary local usage of donor VCAs to a broader exchange of these grafts. This will allow better matching of donors and recipients, thereby improving functional and cosmetic short- and long-term results of transplantation. Establishing such a structured and transparent allocation system and further improving outcomes of VCA transplantation will foster general acceptance of this type of transplantation, which could have a further positive effect on VCA donation rates.
